# Seasonal toxicity of urban road dust in runoff process-studies in Poland

**DOI:** 10.1007/s11356-024-33716-w

**Published:** 2024-05-28

**Authors:** Justyna Rybak, Zbigniew Ziembik, Magdalena Wróbel, Jan Stefan Bihałowicz, Wioletta Rogula-Kozłowska, Niranjala Dissanayake Mudiyanselage, Grzegorz Majewski

**Affiliations:** 1 Faculty of Environmental Engineering, Wrocław University of Science and Technology, Wybrzeże Wyspiańskiego 27, 50-370 Wrocław, Poland; 2 Institute of Environmental Engineering and Biotechnology, University of Opole, 6a Kominka Str, 45-032 Opole, Poland; 3 Fire University (former The Main School of Fire Service), 52/54 Słowackiego Str, 01-629 Warsaw, Poland; 4 Institute of Environmental Engineering, Warsaw University of Life Sciences, 02-787 Warsaw, Poland

**Keywords:** Urban road dust, Toxicity, Elements, Bioindicators, Runoff, Seasons

## Abstract

Urban road dust (URD) is one of the most important non-point sources of pollution in agglomerations. The aim of this study was to assess the seasonal toxic effects of URD runoff in two regions of Poland. The concentrations of elements in URD and leachate were studied. The impact of pollutants in URD runoff on water organisms was evaluated using Daphtoxkit F and Rotoxkit F (LC50). The acute toxicity tests for crustaceans and rotifers were selected as the response of these taxa reflects the impact on zooplankton, a key component of aquatic ecosystem and the basis of most food webs. The concentrations of elements were found to vary depending on the site, although URD samples collected in Katowice agglomeration (Upper Silesia) had higher values of elements (Mn, Cu, Zn, As) compared to Wrocław (Lower Silesia). The concentrations of Mn, Zn, As, Cr, and Mg in water-soluble fraction of URD were higher in summer and winter in the Upper Silesia region due to rainwater runoff resulting from traffic, industries, post-industrial waste, and the presence of old heating systems. When comparing the content of elements in the water-soluble fraction between seasons, Zn, As, Cr, and Al concentrations were slightly higher in winter. The highest mortality of *Daphnia magna* and *Brachiouns calyciflorus* was observed in URD from both agglomerations in winter. However, the mortality is likely due to the concentration of elements or/and the coexistence of an unknown compound or a synergistic effect of the studied elements. This study highlights the alarming seasonal sources of elements in URD runoff, which will directly enter the food chain and affect the entire ecosystem, and human health.

**Supplementary Information:**

The online version contains supplementary material available at 10.1007/s11356-024-33716-w.

## Introduction

Pollution is one of the most important environmental problems in Poland, and it has become an important issue worldwide (Hanke et al. 2022). Big cities such as Wrocław (Lower Silesia) and Katowice (Upper Silesia) have reported significantly higher pollution levels (Górka et al. 2020; Kobza et al. 2018). In Wrocław agglomeration, in addition to low emissions, pollutants of traffic origin still tend to dominate despite evolving mobility policies (Koczan 2021). In Katowice agglomeration, pollutants are primarily of industrial and municipal origins, where exceedances of standards are often encountered (Góra 2020), which leads to work on modelling and forecasting of pollution (Foszcz et al. 2021). Thus, the complex problems and consequences of anthropogenic activities lead to the formation and movement of pollutants that vary in composition (Kowalska 2020). The most dangerous pollutant is tiny dust particles because the metals attached to them have toxic effects and affect the entire ecosystem (Suvetha et al. 2022). Urban road dust (URD) is one of the most important non-point sources of pollution in urban sites (Penkała et al. 2023). Potentially harmful contaminants found in URD come from a variety of anthropogenic sources, including erosion of surrounding soil, atmospheric deposition, and anthropogenic activities, including traffic-related activities (Krupnova et al. 2020). URD comprises primarily soil-derived minerals, such as quartz, albite, muscovite, chlorite, and microcline and organic matter from plant matter, such as mould spore, pollen, and animal skin remains. In addition, 30% of URD consists of toxic pollutants of brake and tyre wear, combustion emissions, fly ash, and heavy metals such as Cd, Cr, Zn, Cu, Ni, and Pb, which are derived from vehicle traffic, and Mn, Fe, and Al, formed from surrounding soils (Khanal et al. 2014; Rogula-Kozłowska et al. 2014; Suryawanshi et al. 2016). There are also studies that indicate the presence of microplastics in URD (Wang et al. 2022). The composition, although similar in many places, is nevertheless unique and is conditioned by the prevailing surrounding conditions in a given location at a given time (Vlasov et al. 2022). As URD can occur in three states (i.e., solid, liquid, and gaseous), it can be particularly dangerous to the environment because it can enter surface waters through runoff. When it contaminates water, it can have a toxic effect on the entire aquatic biocenosis, because runoff that can be described as urban in origin can have high enough concentrations (also exceeding standards) of pollutants that can severely harm living organisms (Hwang et al. 2016a, b). The runoff process depends on the amount of precipitation, and toxicants are divided between the wet road dust and the leachate (Khanal et al. 2019). Toxic polar and non-polar substances dominate in the wet phase, although pH-dependent and water-exchangeable toxicants are predominant in the leachate (Watanabe et al. 2011). As toxicants are loosely bound in the wet phase, they could migrate into leachate increasing its toxicity. On the other hand, the amount of precipitation can also dilute the leachate. However, strongly bound toxicants are also present in the wet road dust. Therefore, the toxicity of this phase can be strong despite the migration of some toxicants. Thus, it is necessary to evaluate the toxicity of URD to aquatic species, so that a basis can be found for broader regulation within the context of transportation or emissions.

Another important aspect is that the composition of URD and the volume of runoff containing these pollutants can vary from season to season. According to Gustafsson et al. (2019), there is a seasonal fluctuation, with early autumn and late spring seeing the lowest loads (minimum of 15 g/m^2^) and late winter and early spring seeing the highest loads (maximum of 200 g/m^2^). The characteristics of URD of the summer season differ from those of the winter season. Summers in Poland become anomalously warmer and drier, albeit with temporary heavy rains and thunderstorms. The air in cities is mostly draughty, and pollutants are deposited on infrastructure. Winters, on the other hand, while no longer an example of full snow and ice, but rather rain or rain with snow, are still very common with temperature variation passing 0 ℃. Furthermore, the composition and the load of the URD vary between streets and depend on the properties of the pavement surface. Thus, road salt is still the most commonly used road and sidewalk maintenance aid. Due to variations in the rates of suspension from various parts of the road surface, the dust load varies greatly between streets, with low amounts in wheel tracks and greater amounts in-between and outside the tracks (Denby et al. 2014; Gustafsson et al. 2019). Thus, in winter, there is more mud or temporary snow on the roads, and consequently, dust contains salt and other materials that are applied to road surfaces. In winter, there is an increase in the usage of vehicle parts (e.g., wear on brake pads or increased degradation of rubber in tyres) and problematic fuel consumption issues through altered engine operation at lower temperatures, which can also directly and indirectly alter the composition of dust from urban roads (Gustafsson et al. 2019). Although, by definition, industrial or heating combustion products alone are not directly responsible for URD, it should be mentioned that during the winter, they strongly increase the amount of pollution that comes from the air into the soil and water. However, very few studies relate URD composition values to specific seasons and conditions during them. Most studies evaluated the runoff toxicity on benthic organisms (Carrasco-Navarro et al. 2022; Gillis et al. 2022; Khanal et al. 2019; Kunz et al. 2022; Shenton et al. 2022; Watanabe et al. 2011, Watanabe et al. 2013). There are a few, mainly old, studies on road runoff water using bacteria and other pelagic species (Greenstein et al. 2004; Kayhanian 2010; Kayhanian et al. 2008; Schiff et al. 2003). However, they focus on marine aquatic life (Greenstein et al. 2004; Schiff et al. 2003), three freshwater species (water flea *Ceriodaphnia dubia*, fathead minnow *Pimephales promelas*, and green algae *Pseudokirchneriella subcapitatum*) and the luminescent bacteria *Photobacterium phosphoreum* using Microtox™ (Kayhanian 2010; Kayhanian et al. 2008). The toxicity of various products used for winter road maintenance on the freshwater biota mayfly *Colobruscoides giganteus* (Ephemeroptera: Colobruscoidea) was assessed by Moulding et al. (2022).

Although from the observational perspective it seems necessary, we are not aware of any studies in which the authors have tested the toxicity of URD on freshwater organisms in the runoff process while taking into account the seasonality of URD and comparison of two polluted cities differed with the origin of pollution. This study undertakes a comparison of samples from two large polluted cities in southern Poland under different conditions (two seasons) and takes into account the diversified origin of pollution to satisfy the above-mentioned research gap.

Therefore, the aim of the study was to assess the seasonal toxic effect of URD runoff in two regions (i.e., Lower and Upper Silesia).

As surface runoff studies have not been comprehensively examined in terms of seasonality and on several types of aquatic organisms, i.e., using “a battery of tests,” on the basis of these studies, it is possible to determine whether such runoffs pose a threat to the aquatic organisms, whether there is an impact of seasonality on toxicity, and, what is the most important, to draw broader conclusions regarding the toxicity patterns of surface runoff toward water organisms.

For this reason, the collected samples were tested for elemental concentrations (i.e., Mn, Ni, Cu, Zn, As, Cr, Mg, and Al) and their effects on living organisms using two commercial ecotoxicity tests, Daphtoxkit F and Rotoxkit F. The selected organisms are commonly used because of the following characteristics: they have adequate sensitivity to a wide range of pollutants; they are ecologically very important components of the aquatic environment, which is an integral part of the studied cities; and they are exposed to ever-increasing concentrations of hazardous metals and metalloids and increasing amounts of runoff (in this study, we used the simulated runoff prepared in the laboratory). Hence, it is necessary to constantly monitor surface water using such organisms in areas suspected of increased contamination by URD and other pollutants migrating between air, soil and water.

## Materials and methods

### Sampling

**Fig. 1:**
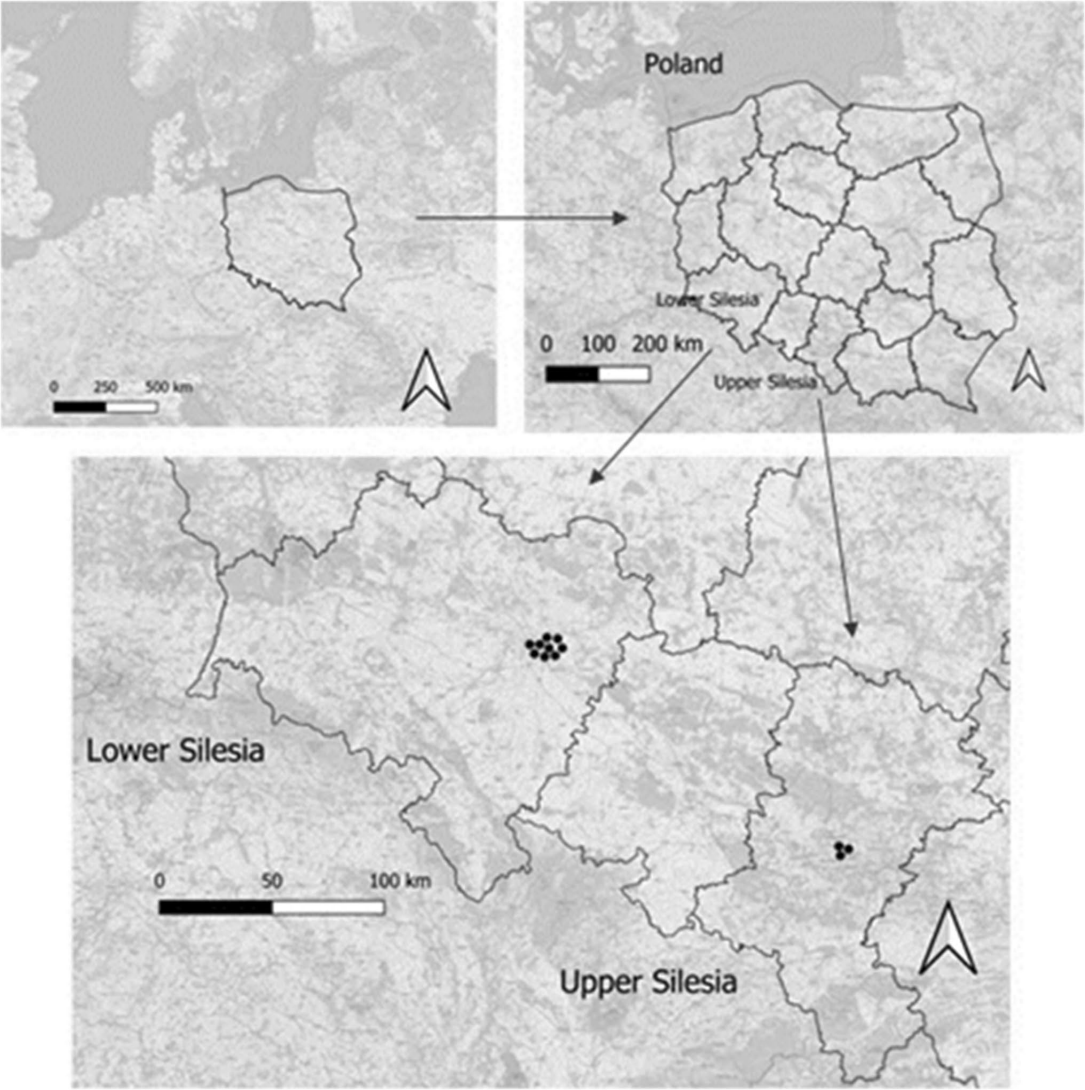
The area of studies with sampling points marked on map of Lower and Upper Silesia in Poland

The research was conducted in and around the agglomeration of Wrocław (WRO1–WRO9) and in and around the agglomeration of Katowice (KAT1–KAT3). In both regions, sampling included city centers and suburbs and small villages. The reason for selecting these regions was the following: they are one of the largest cities in Poland, including significantly different sources of pollution, the first where traffic pollution dominates and the second with prevalence of industrial pollution. Samples were taken into account based on three important criteria. The intensity of car traffic and, the road proximity was considered the most crucial, followed by attention to the proximity of industrial facilities or heating type. The final criterion was the type of surroundings with the proximity of green areas. A detailed description was presented in Fig. 1 and Table S2 in Supplementary material.

The samples were collected in June 2018 in the summer season and in February 2019 in the winter season. Sampling was conducted with a vacuum cleaner. The empty pre-weighed filter bag was attached to a portable vacuum cleaner, to gather URD from road surfaces. Every research site was distinct from the others, having unique characteristics pertaining to the surrounding neighborhood type and its unique sources of pollution. Six sub-samplings of a single site were collected in order to rule out the possibility that the URD was a random sample and to ensure that it is representative for a wider region than the sampling point alone. For sites that were close to roadways, we collected three samples on one side and three more samples on the other. The separation between the spots was roughly 20 m. When it came to intersection sites, we also collected six samples: two samples were taken close to the center, and four samples were taken at the intersection’s corners.

Then, the URD was weighed, dried, and sieved in order to remove larger impurities. Samples were split into two parts for to total metal content analysis and to prepare water extracts. Then, 30 g of the URD was used for the preparation of water extracts for toxicity tests. According to Watanabe et al. (2013), toxic elements from URD that were washed off by sudden precipitation had higher toxicity than wet road dust samples. Water extracts of the URD samples (simulated runoff) were prepared according to the previously described procedure (Rogula-Kozłowska et al. 2021; Watanabe et al. 2013). To obtain the water extracts, the URD samples were dried and then mixed with water in a ratio of 1:2. The next step was to centrifuge the samples according to the procedure (Watanabe et al. 2013). Then, the samples were shaken for 12 h, and the pH was measured in the water extracts with a pH meter (Table S1 in Supplementary material). The remaining prepared water extracts of URD were refrigerated until water-soluble (WS) metals analysis. The other part of every collected sample was dried and stored until the total metal analysis was conducted. The detailed site description is presented in Table S2. Figure 1 shows the areas studied.

### Elemental analysis

The natural content of trace elements in the environment, which is a derivative chemical composition of the parent rock, geological and soil-forming processes, generally does not pose a threat to living organisms. However, under conditions of strong anthropopressure, the content is excessive. Therefore, in our studies, we focused on the assessment of the following elements: Mn, Cr, Ni, As, and Cu. These elements are connected with industrial activity, such as smelters, and/or are classified as elements with a very high or high degree of potential hazard that can pose a threat to living organisms. Our studies also assessed other elements, such as Al, Mg, and Zn, which are the most abundant elements in the Earth’s crust. What is more, Cr, Cu, Mn, Ni, and Zn have been classified as environmental priority pollutants by the United States Environmental Protection Agency (US EPA 2014). URD samples were analyzed for elements according to EPA Method 3051 and Method 200.8 (Revision 5.4; US EPA 2009; US EPA 2007). The analyses included WS metals (water extracts) and total metal (in URD) assessments. The first stage to determine the metal composition of URD was drying the samples of URD at 50 ℃ for 48 h and then sieving them to remove coarse debris. Subsequently, the weighed samples (about 0.3–0.7 g) were digested in a mixture of 3 cm^3^ of HNO_3_ (65%) and 1 cm^3^ of HF (40%) for 6 min. After the water extracts were prepared as described above, they were also analyzed. Simultaneously, blank samples were prepared and analyzed with the element samples. Element concentrations in the blank samples were taken into account in the calculation of the concentrations of the studied samples. The concentrations of Mn, Ni, Cu, Zn, As, Cr, Mg, and Al were assessed with ICP-MS (Elan 6100 DRC-e Perkin Elmer) under the following operating conditions: ICP RF power, 1125 W; nebuliser gas flow rate, 0.78–0.83 L/min; auxiliary gas flow, 1.15 L/min; plasma gas flow, 15 L/min; and sample flow rate, 1 mL/min. The samples were measured in triplicate. The certified multi-element standard stock solutions, Periodic Table Mix 1 and Transition metal mix 2 (Fluka), were applied as calibration solutions. The validation of the method was two certified reference materials, SRM 1643e (recovery in the range 95 Ni–116% Cr) and SRM 1648a (recovery in the range 75 As–127% Cr), obtained from the National Institute of Standard and Technology. Detection limits were as follows: 0.151 µg/L for Al, Mg, and Zn; 0.019 µg/L for As; 0.013 µg/L for Cr; 0.022 µg/L for Mn; 0.017 µg/L for Ni; and 0.048 µg/L for Cu.

The solubility of each element was calculated using the following formula:

$$S=\frac{{C}_{\text{X},WS}}{{C}_{\text{X},T}}\cdot 100\%$$

where $S$ represents the solubility (%),$\text{X}$ represents the element,${C}_{\text{X},WS}$ represents the concentration of the WS fraction of element X (mg kg^−1^), and ${C}_{\text{X},T}$ represents the total concentration of element X (mg kg^−1^).

Solubility results are provided in Table S3 and S4 in the Supplementary material.

### Ecotoxicological tests

The acute toxicity tests for crustaceans and rotifers were selected as the response of these taxa reflects the impact on zooplankton, a key component of aquatic ecosystem and the basis of most food webs. In the previous studies, *Daphnia magna* (crustacean) acute test demonstrated greater sensitivity to Zn, Mn, and Cd than the bacterial assays (Teodorovic et al. 2009). Numerous studies proved the great sensitivity of *D*. *magna* and *Brachiouns calyciflorus* to Cu, Ni, Zn, Mn, As, and Cr (e.g., Couillard et al. 1989; Münzinger and Monicelli 1991; Traudt et al. 2017).

#### Daphtoxkit F magna

The acute toxicity test with *D*. *magna* crustaceans was performed using a test developed by MicroBiotest Inc., Belgium (Daphtoxkit F). The first step of the test procedure was to prepare a standard medium containing concentrated salt solutions and distilled water. The process involved transferring the salt solutions to a 2-L flask, then filling the flask with distilled water to the level of 2 L and aerating the mixture for at least 15 min. Then the contents of the vial with eggs were transferred to a Petri dish and 10 mL of previously prepared medium was added to it. The next step was to incubate the organisms for 3 days before the actual toxicity test. Incubation lasted for 72 h at a temperature of 20–22 °C under continuous lighting of 6000 lx.

Subsequently, a dilution series of the water extract of the URD (100%, 75%, 50%, 25%, 12.5%, and 6.25%) was prepared, and the pre-feeding of the test organisms was carried out. The next step was to fill the test wells with standard medium (10 mL) and appropriate concentrations of extracts and, using a microscope, transfer five test organisms to each well. The test was performed in four replicates, and the standard medium was used as the control solution. The organisms were then incubated at 20 ℃ in the dark. The dead specimens of each dilution of URD water extract were counted after 24 h of incubation.

#### Rotoxkit F

The toxicity test using *B*. *calyciflorus* rotifer was performed as previously described using a screening test developed by MicroBiotest Inc., Belgium (Rotoxkit F). In the first stage of the test, as in the case of Daphtoxkit F magna, the preparation of a standard medium involved creating a mixture of concentrated salt solutions with distilled water, similar to the previous test. Next, the breeding of organisms was prepared which consisted of incubating the eggs with the standard medium for 16 to 18 h at 25 ℃ under continuous lighting of 3000 to 4000 lx. Then, the same dilution series of the water extract of the URD as previously described was prepared, and the organisms were pre-fed. The following steps were the same as in the case of Daptoxkit F. Then, each specimen was transferred to each well and incubated at 25 ℃ in the dark for 48 h. The test was performed in eight replicates, and the standard medium was used as the control solution. After the incubation time, the live organisms were counted.

#### Statistical analysis

The elemental composition of URD and its leachates was analyzed using the metric multidimensional scaling method (Becker et al. 1988; Borg et al. 2013). MDS enables presentation of similarities between objects using distances between pairs of them. A matrix of distances between pairs of points in a multidimensional space of concentrations was constructed. The Aitchison distances between points in an eight-dimensional space were calculated (Aitchison 2003).

Preserving restrictions coming from sample space properties the MDS results enable estimation of the analyzed data structure in multidimensional, Euclidean space. For estimation of the concentrations structures in URD at different places, the 2D plane was selected.

To analyze the results, a generalized linear mixed-effects model (GLMM) was used (Jiang and Nguyen 2021; Nelder and Wedderburn 1972). Fixed effects represent population parameters that are considered constant at all factor levels, while random effects describe group-specific variability (Faraway 2016; Fox and Weisberg 2018). The data analysis focused on the chemical element concentration (a numerical variable) and the area of sample collection (a grouping variable, agglomeration name: Wrocław or Katowice) as fixed explanatory variables. The random effect was represented by the sampling sites. The response variable was the logit function of the species mortality. Separate models were constructed for each chemical element. The model’s estimators of structural parameters, specifically the intercept ${\beta }_{0}$ and slope ${\beta }_{1}$, were calculated. Additionally, the differences in intercept ${\beta }_{2}$ and slopes ${\beta }_{3}$ between both agglomerations Wrocław and Katowice were calculated along with the $p$ values for the null hypothesis ${H}_{0}$: ${\beta }_{i}=0$ for $i\in \left\{\text{0,1},\text{2,3}\right\}$. To estimate covariabilities between the element concentrations (T) in URD and leachate (WS), the Spearman correlation coefficients *r*_*S*_ between concentrations in summer and winter and their $p$ values (*p*_*S*_) were calculated.

The impact of summer and winter road dust on organisms was compared using the Wilcoxon signed rank test (real statistics using excel: https://real-statistics.com/ n.d.; Wilcoxon 1945). The test was performed on the reciprocal of the $LC$ i.e. ${\left(LC\right)}^{-1}$ to correctly rank points where $LC$ was not determined. In such cases it was assumed that the reciprocal of $LC$ tends to zero. The alternative hypothesis ${H}_{1}$ stated that toxicity ranks are higher in winter than in summer. The concentrations of metals were analyzed using principal components analysis (PCA) for both regions. PCA was proved to be a versatile tool in analyzing, predicting, and understanding air pollution dynamics. This tool is utilized in various studies for source apportionment (Cesari et al. 2016). For each season, the principal components along the sites were identified. The cosine similarity between the PCA components was analyzed.

The lethal concentration $L{C}_{50}$ was calculated to study toxicity. $L{C}_{50}$ is the concentration that causes a lethal effect in 50% of the tested specimens, calculated statistically from water extract. The higher the value of $L{C}_{50}$ the less toxicity was recorded. The calculations were based on a logistic regression model.

## Results and discussion

### Elemental analysis

The total content of elements in URD in the summer and winter seasons is presented in Tables 1, 2, S5 and S6, which show the detailed data concerning element concentration in URD at the sampling sites. The total and WS fraction are included in Tables S5–S8 in the Supplementary material. The concentrations of the elements in URD vary depending on the sampling site. Sites KAT 1, KAT 2, and KAT 3 are located in industrial areas near busy roads. Therefore, much higher values of elements such as Mn, Cu, Zn, and As were observed in URD from industrial areas than at sites in the Wrocław agglomeration (i.e., Lower Silesia). Cu is used to improve the corrosion resistance and strength of car parts (Kim et al. 2021), while Zn can come from the wear of car tyres and from the corrosion of galvanised car parts (Jeong 2022). The presence of Mn is connected with petrol combustion (Tümer et al. 2022). Mining, smelting, and burning of fossil fuels are the major industrial processes that contribute to As contamination (Briffa et al. 2020). The concentration of the total element content in URD in Upper Silesia was higher in winter than in summer. Elements such as Zn, Cr, Al, and Mg in URD had particularly higher concentrations in winter. The origin of Cr in URD could be industrial, although Cr could be present through the corrosion of parts of vehicles, which could be particularly severe in winter (Gustafsson et al. 2019; Lu et al. 2009). Ca and Mg are common components of building cement used for road and bridge construction, which may affect the content of both elements in URD. In this study, the content of Ca was not assessed, but the high content of Mg suggests its origin from construction materials, even though this element can also be related to the movement of vehicles and coal combustion (Skorbiłowicz and Skorbiłowicz 2019). These findings suggest a greater influence of urban transport, existing industries, and post-industrial waste but also old heating systems could contribute highly to the toxicity of URD. As it was mentioned in Table S1 near studied sites in Katowice agglomeration, single low-rise building with individual old heating system predominates. Burning coal and wood in boilers to heat residences is emitting excessive pollution, especially old-type boilers, which are characterized by high emissions (Mahmoud et al. 2021).

**Table 1:** Total concentration ${C}_{\text{X},T}$ (T) of elements in URD at sampling sites in summer (mg kg^−1^)

${C}_{\text{X},T}$	Mn_T	Ni_T	Cu_T	Zn_T	As-T	Cr_T	Mg_T	Al._T
Upper Silesia								
Min	1040	29	187	3201	136	43	15,600	8026
Max	2984	59	458	9535	405	64	43,600	12,2248
Median	1950	33	284	4076	191	61	29,300	9378
Mean	1991	40	310	5604	244	56	29,500	46,551
Standard deviation	794	13	112	2802	116	9	11,432	53,529
Lower Silesia								
Min	171	30	38	84	2	39	4290	3912
Max	424	102	483	230	5	206	11,600	9159
Median	248	47	69	140	3	59	5830	5023
Mean	258	50	126	153	3	77	6700	5133
Standard deviation	107	31	206	60	1	75	3158	2302

**Table 2:** Total concentration ${C}_{\text{X},T}$(T) of elements in URD at sampling sites in winter (mg kg^−1^)

${C}_{\text{X},T}$	Mn-T	Ni-T	Cu- T	Zn-T	As-T	Cr-T	Mg-T	Al-T
Upper Silesia								
Min	70	3	6	5780	0	8	32,500	14,000
Max	1024	39	156	11,240	815	390	83,600	245,230
Median	772	29	47	6340	2	66	45,140	16,540
Mean	622	24	70	7787	273	155	53,747	91,923
Standard deviation	403	15	63	2453	384	168	21,731	108,409
Lower Silesia								
Min	174	17	15	238	2	31	4830	6394
Max	310	227	41	451	3	159	11,400	10,503
Median	203	44	28	388	2	54	5440	8247
Mean	223	83	28	366	2	74	6778	8348
Standard deviation	58	93	10	89	1	56	2972	1680

The total average URD concentration of Zn for the Katowice agglomeration in summer was 5604 mg kg^−1^, while it was 7787 mg kg^−1^ in winter. For Al, the concentration was 46,551 mg kg^−1^ in summer and 91,923 mg kg^−1^ in winter. The total average URD concentration in the Wrocław agglomeration also varied between seasons, although not very highly. For example, the average Zn concentration was 153 mg kg^−1^ in summer and 366 mg kg^−1^ in winter. For Al, it was 5133 mg kg^−1^ in summer and 8348 mg kg^−1^ in winter.

The possible reasons for general higher levels of metal concentration in URD during winter is linked to the annual cycle which leads to expanding URD sources and retaining dust in the fall and winter. The opposite conditions lessen the amount of URD in the spring (Gustafsson et al. 2019).

According to the International Agency for Research on Cancer, As, Cr, and Ni compounds are classed as human carcinogens. According to recent reports, Cr, Ni, Cu, and Zn support electron exchange and help the sun create reactive oxygen species (Morakinyo et al. 2021). Because of their extended residence period in the environment, persistent bioavailability, and toxicity even at low concentrations, these elements are considered environmentally significant components. What is more, previous epidemiological researches have shown a correlation between cardiovascular disease and exposure to high concentrations of certain metals, such as Ni, Cu, and As (Moradnia et al. 2024; Morakinyo et al. 2021). For this reason, the high content of these metals in URD, particularly in winter, is disturbing and clearly proves that in large cities in Poland there is a high risk of these metals entering the water environment and their impact on the aquatic organisms.

This study documents seasonal pollution in water. Any chemicals and URD related with road runoff can be swept during rain events or snowmelt to waterbodies. To characterize exposure and evaluate the potential risk to aquatic organisms, it is necessary to comprehend the transport and fate of road-associated contaminants, including their re-distribution through air to soil environment and wash-off into water (Chen et al. 2016). URD runoff can deposit contaminants on surfaces, bioaccumulate them into sediments, or dissolve in surface waters. The type of substances and their reactivity in water will affect exposure level of organisms. The content of the WS fraction in URD in summer and winter is presented in Tables 3, 4, S7 and S8 (Supplementary material). Similarly, the concentrations varied depending on the sampling site. The highest values were recorded for the average content of Mn, Zn, As, Cr, and Mg in summer and winter in the Upper Silesia region compared to the values for Lower Silesia. These observations suggest that the leachate from Upper Silesia can be characterized by a higher content of soluble elements and indicate a good solubility of the chemical elements, and therefore their impact on aquatic organisms. Runoff pollutants from URD can significantly impact the sediments of surrounding waters. The fractions of elements such as as Zn, As, Cr, Mg, and Al were higher in winter, than in summer in Upper Silesia. The exception were Mn, Ni, and Cu as these elements were higher in summer. On the other hand, in Lower Silesia, all elements (Mn, Ni, Zn, Cr, MG, and Al) were higher in winter with the exception of Cu and As.

**Table 3:** Elemental concentrations in water soluble ${C}_{\text{X},WS}$ (WS) URD fraction at sampling sites in summer [mg kg^−1^]

${C}_{\text{X},WS}$	Mn-WS	Ni- WS	Cu-WS	Zn-WS	As-WS	Cr-WS	Mg-WS	Al-WS
Upper Silesia								
Min	44	8	4	17	21	3	15,406	4
Max	2540	11	14	45	31	11	42,433	5
Median	578	8	9	19	28	9	28,727	4
Mean	1054	9	9	27	27	8	28,855	4
Standard deviation	1073	1	4	13	4	4	11,034	1
Lower Silesia								
Min	1	4	5	2	1	2	2839	1
Max	196	12	58	12	4	14	5642	7
Median	34	7	21	6	3	4	3711	4
Mean	57	7	23	6	3	5	3906	4
Standard deviation	86	3	22	4	1	5	1174	3

**Table 4:** Elemental concentrations in water soluble ${C}_{\text{X},WS}$ (WS) URD fraction at sampling sites in winter [mg kg^−1^]

${C}_{\text{X},WS}$	Mn-WS	Ni- WS	Cu-WS	Zn-WS	As-WS	Cr-WS	Mg-WS	Al-WS
Upper Silesia								
Min	15	1	0.4	11	0.1	0.3	29,413	6
Max	633	6	2	57	118	50	77,246	11
Median	38	5	1	23	0.1	14	42,161	8
Mean	229	4	1	30	39	21	49,607	8
Standard deviation	286	2	0.5	19	56	21	20,225	2
Lower Silesia								
Min	0.2	3	1	1	1	1	3547	0.1
Max	266	50	56	18	7	14	5764	14
Median	39	5	8	10	2	3	3993	7
Mean	70	11	17	9	2	5	4269	7
Standard deviation	119	22	24	7	3	6	961	6

For the WS-fraction in summer, the highest values were recorded in URD collected at the Katowice agglomeration in the case of Mn, As, Zn, and Mg, which suggests that the amount of simulated runoff from that region can have great impact on water organisms.

It can also be stated that although the concentration in URD of studied elements is higher in the Katowice region, specific patterns of the frequency of occurrence are more similar in summer among sites for both regions and differ in winter (Tables 3 and 4). In general, the elements concentration in summer was in order: Mg > Mn > As > Zn > Cu > Ni > Al for Upper Silesia and Mg > Mn > Cu > Ni > Zn > Cr, Al > As, and in winter Mg > Mn > As > Zn > Cr > Al > Ni > Cu for Katowice agglomeration and Mg > Mn > Cu > Ni > Al > Zn > Cr > As for Wrocław agglomeration.

Conferring to European Environment Agency (EEA), as a whole in Europe, heavy metal emission was declined since 2005. Although in 2021, Germany, Italy, and Poland contributed most to heavy metal emissions (EEA, 2023a). World Health Organization (WHO) issued air quality guidelines (AQGs) and estimated reference levels (RLs) in the air (EEA, 2023b). As there are no such guidelines regarding the content of specific metals in URD, and in surface runoff the obtained values in our study were compared with the literature data (Tables 5 and 6). The content of selected elements in URD in other sites worldwide is presented in Tables 5 (total content) and 6 (WS fraction). Values in Table 5 indicate that Mn-T (T-total concentration), Zn-T, Mg-T, and Al-T concentrations in URD in the Katowice agglomeration were the highest in relation to other records. Such a result suggests the high impact of anthropogenic emission sources such as industrial emissions. Therefore, the amount of metals in URD deriving from traffic or industrial emissions could be used as proxy for potential human health impacts.

**Table 5:** Comparison of the total element concentration in URD in Poland with selected places in the world (average values) (T-total) [mg kg^−1^]. Symbols w or s mean summer or winter as the maximum values were presented

Country	City	Mn-T	Ni-T	Cu-T	Zn-T	As-T	Cr-T	Mg-T	Al-T	References
Poland	Katowice aggl	2984s	59s	458s	11240w	815w	390w	83600w	245230w	This study
Poland	Wrocław aggl	424s	227w	483s	451w	5s	206s	11600s	10503w	This study
Greece	Thessaloniki	529s	96s	526s	671s	13s	187s	9500s	29200s	Bourliva et al. (2016)
Australia	Sydney	48s	15s	160s	850s	–	65s	–	–	Birch and Scollen (2003)
Turkey	Tokat	285s	65s	29s	63s	–	30s	–	–	Tuzen (2003)
England	New Castle	–	26s	132s	421s	–	–	–	–	Okorie et al. (2012)
Iran	Teheran	1176s	31s	203s	791s	–	31s	–	–	Saeedi et al. (2012)

**Table 6:** Comparison of the water-soluble fraction of elements in URD in Poland with selected places in the world (average values) (WS—water-soluble) [%]. Symbols w or s mean summer or winter as the maximum values were presented

Country	City	Mn	Ni	Cu	Zn	As	Cr	Mg	Al	References
Poland	Katowice aggl.	52s	27s	17s	3s	31w	15w	86w	11w	This study
Poland	Wrocław aggl.	26w	16w	27s	4w	82w	8w	65w	14w	This study
Greece	Thessaloniki	12w	15w	10w	10w	90w	30w	30w	70w	Voutsa et al. (2015)
China	Beijing	30w	30w	20w	40w	40w	10w	–	2w	Feng et al. (2009)
Hungary	Tihany (Lake Balaton)	5s	32s	13s	12s	19s	17s	–	–	Hlavay et al. (1998)
UK	Edinburgh	38s	10s	45s	60s	63s	13s	–	–	Heal et al. (2005)

**Fig. 2:**
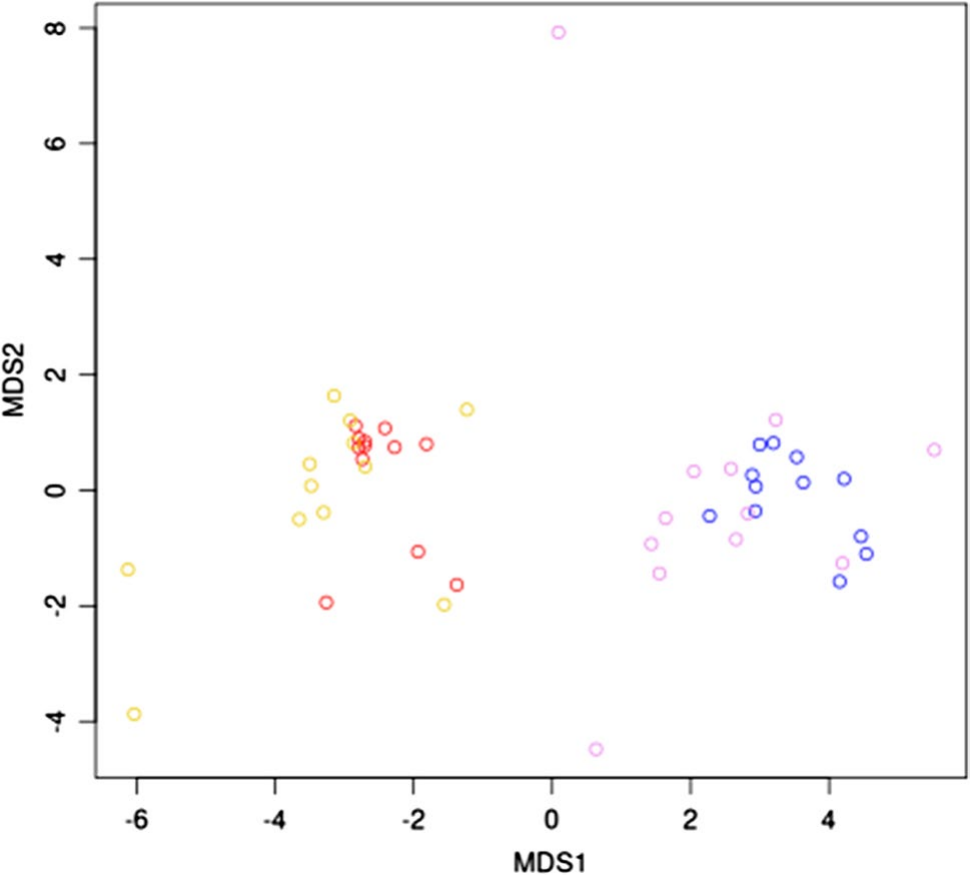
MDS distances between points representing chemical compositions of URD and leachates. Red and gold points represent URD composition in summer and in winter respectively. Composition of leachates is marked with violet (winter) and blue (summer) points. Two groups of points separated from each other means a clear division in both agglomerations in URD composition

Figure 2 shows the results of the principal coordinates analysis (MDS). A matrix with 2 columns, MDS1 and MDS2, was calculated. Rows of the matrix give the coordinates of the points chosen to represent the dissimilarities. Two groups of points can be observed (Fig. 2). One represents URD composition, and the second shows the composition of leachates. No substantial differences were observed between composition in summer and in winter. However, regarding the sampling areas, the groups of points for Wrocław and Katowice agglomerations were clearly separated. This observation indicates a different composition of URD in both regions in respect of the determined elements.

Additionally, principal components were determined according to maintaining > 95% of the explained variance out of the original data of the total content of elements (Table 7). Three and four principal components were identified for summer (PCxs) and winter (PCxw), respectively. Additionally, the cosine similarity between principal components in summer and winter was calculated (Table 8). Based on the results, we can conclude that PC1s and PC1w are very similar and can correspond to the same source of pollution. These components are strongly related to the Mn, Zn, Mg, and Al and what is differing them from other PC is Mn. Mn is a signature for the brake wear and, in general, steel products as well as fuel additives (Tümer et al. 2022). This shows that PC1s and PC1w are components related to road traffic that is rather independent on the season. Since components are ordered by eigenvalue, and PC2s and PC3w are significantly correlated; therefore, this finding suggests that in winter, the additional component PC2w, which is weakly or negatively correlated with any PCxs, appears. PC2s and PC3w are related to Zn, Mg, and Al, which are mainly connected to combustion processes (traffic origin) and industrial and construction activities. Zn is also a terrigenous marker associated with mineral particles in soil (González-Miqueo et al. 2009). Additionally, PC2s include Cu and As, which indicate higher participation of industrial emissions in summer. PC2w is related to Al, Ni, and Cr, which can be present due to industrial activity, the use of liquid and solid fuels and municipal waste (Gustafsson et al. 2019; Lu et al. 2009; Pernigotti et al. 2016). What is important is that the cold season increases the risk of corrosion due to high exposure to salt and water (Gustafsson et al. 2019). Therefore, the participation of these elements in URD is probably higher than in summer. PC4w includes As, which is related to anthropogenic sources (Kumar et al. 2013).

**Table 7:** The principal components for summer (PCxs) and winter (PCxw) total concentrations ${C}_{X,T}$ of studied elements

Element X	PC1s	PC2s	PC3s	PC1w	PC2w	PC3w	PC4w
Mn	− 0.4127	− 0.0031	− 0.1141	− 0.4520	− 0.1055	− 0.2670	− 0.0440
Ni	− 0.2952	− 0.5466	0.0276	0.0735	0.3604	− 0.7616	0.3115
Cu	− 0.2618	0.1014	0.9576	− 0.0769	− 0.5729	− 0.3204	− 0.6217
Zn	− 0.4106	0.0898	− 0.1222	− 0.4435	− 0.0246	0.2566	0.2953
As	− 0.4101	0.0922	− 0.1321	− 0.1554	− 0.5907	− 0.2517	0.5261
Cr	− 0.0136	− 0.8117	0.0444	− 0.3789	0.3520	− 0.2839	− 0.2833
Mg	− 0.4108	0.0900	− 0.1222	− 0.4698	− 0.0383	0.1545	0.1655
Al	− 0.4102	0.0865	− 0.1410	− 0.4464	0.2364	0.1112	− 0.2072
Total explained variance	72%	91%	99%	53%	76%	90%	98%

**Table 8:**
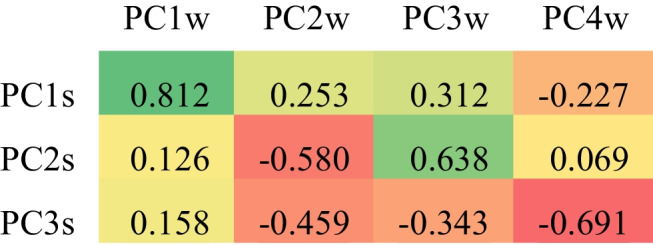
The cosine similarity between principal components in summer and winter

### Ecotoxicological tests

Ecotoxicity tests were performed to assess whether URD can be toxic to water species. The test results (Daphtoxkit F magna and Rotoxkit F) showed the toxicity of URD to the studied organisms (Table 9).

**Table 9:** Values of $L{C}_{50}$ (%) for *D*. *magna* and* B*. *calyciflorus* in summer and winter at sampling sites

Sample ID	*D*. *magna*		*B*. *calyciflorus*	
	Summer	Winter	Summer	Winter
Control	–	–	–	–
KAT 1	5.28	4.32	32.77	27.67
KAT 2	14.2	11.4	27.9	18.21
KAT 3	–	–	–	52.87
WRO 1	28.2	21.43	–	62.11
WRO 2	-	78.9	–	63.65
WRO 3	19.23	15.78	19.84	12.65
WRO 4	-	68.27	–	–
WRO 5	9.39	9.32	63	51.87
WRO 6	16.6	15.2	57.8	41.87
WRO 7	33.78	28.15	60	62.1
WRO 8	–	–	–	73.67
WRO 9	–	–	–	–

“–” means that calculations were not possible

#### Daphtoxkit F magna

The highest toxicity to *D*. *magna* ($L{C}_{50}=4.32\%$) was recorded in URD collected from the Katowice agglomeration in industrial areas in winter (KAT 1), and also in URD collected in the Wrocław agglomeration (city center, WRO 5) in winter ($L{C}_{50}=9.32\%$). Higher LC50 values were observed in the summer season, indicating lower toxicity. However, despite the seasonal change, KAT1 and WRO5 samples remained the most toxic, consistent with the winter season.

#### Rotoxkit F

The lowest $L{C}_{50}$ values (i.e., the highest toxicity) were recorded in the URD collected in the center of the Wrocław agglomeration for *B*. *calyciflorus* (WRO 3, $L{C}_{50}=12.65\%),$ and for the industrial area of the Katowice agglomeration (KAT 2, $L{C}_{50}=18.21\%$) in winter and summer (WRO 3, $L{C}_{50}=19.84\%$; KAT 2, $L{C}_{50}=27.9\%$), respectively. A lower or even no toxic effect was noted in the URD samples collected in the suburbs of both studied agglomerations. There was a greater effect of URD collected in winter than in summer. Samples of URD collected in the winter showed lower $L{C}_{50}$ values than in summer. For example, in KAT 2, $L{C}_{50}$ was 28% in summer and 18% in winter.

The results showed a significant difference ($p<0.01$) for $L{C}_{50}$ at all studied sites for *D*. *magna* and *B*. *calyciflorus*, which suggests that URD in winter is more toxic than road dust in the summer. However, this mortality is likely due to the concentration of elements rather than their type. In addition, the combination of different elements in the leakage can have a toxic effect toward water organisms. The coexistence of the unknown compound may also contribute to the mortality of species. What is important, such factors as road salt, which were not studied in this research, can contribute greatly to the aquatic toxicity as significant effects were noted in studies conducted in USA where winter runoff showed chronic and acute toxicity in *Ceriodaphnia dubia* and *Pimephales promelas* bioassays because of presences of road salt (Corsi et al. 2010).

Moreover, recent research has focused on tire wear chemicals N-(1,3-dimethylbutyl)-N′-phenyl-p-phenylenediamine (6PPD) and its derivative 6PPD-quinone, which are thought to be new contaminants. 6PPD is a commonly used antioxidant and antiozonant that is released during the manufacture or usage of products connected to rubber. Rubber-related goods are widely used and produced in large quantities, which has led to the identification of 6PPD and 6PPD-quinone as being widely present in the environment. The majority of current research revealed the presence of 6PPD and 6PPD-quinone in URD and surface water. The toxicological mechanisms are yet unknown, despite the fact that these chemicals may have negative effects on aquatic life and that 6PPD-quinone is toxic to certain species (Chen et al. 2023).

### Estimation of differences in element toxicity between sampling areas

Our research studied the influence of the simulated URD runoff on the test organism. Although the concentration of the selected elements in leachate (WS-elements) was determined, other toxic compounds can possibly be discharged from the URD. If the unknown compound coexists in URD with the element determined, a common influence of the two (or more) components is observed. As a result, different characteristics of the element toxicity can occur.

The GLMM describes changes in the test organisms’ mortality observed for leachates from URD collected in Wrocław and Katowice agglomerations. Table 10 shows intercepts and slopes of the relationship for both areas, and the structural parameters ($\beta$), their standard errors ($SE$), and $p$ values ($p$) for ${H}_{0}$: ${\beta }_{i}=0$ are presented. For the Katowice region, the intercept (${\beta }_{0}$) and slope (${\beta }_{1}$) for the linear relationship were calculated. The ${\beta }_{2}$ parameter represents the difference between intercepts calculated for Katowice and Wrocław agglomerations. For both areas, the difference in slopes is represented by ${\beta }_{3}$.

**Table 10:** The structural parameters ${\beta }_{i}$ ($i\in \left\{0,\dots 3\right\}$), standard errors ($SE$), and $p$ values ($p$) for H0: ${\beta }_{i}=0$ for the GLMM model describing relationship between the element concentrations (WS- fraction) and mortality of the test organisms

*D*. *magna*												
	*β*_0_	*SE*_*β*0_	p_β0_	*β*_1_	*SE*_*β*1_	*p*_*β*1_	*β*_2_	*SE*_*β*2_	*p*_β2_	*β*_3_	*SE*_*β*3_	*p*_*β*3_
Al	− 1.11	1.47	0.448	4.03	0.58	0.000	0.44	1.75	0.803	− 1.15	0.63	0.068
As	2.19	2.24	0.329	3.40	0.56	0.000	− 2.02	2.58	0.434	− 0.34	0.60	0.570
Cr	− 0.53	2.27	0.815	3.81	0.56	0.000	− 1.15	2.62	0.661	− 0.86	0.60	0.150
Cu	2.88	1.46	0.049	3.90	0.57	0.000	− 4.63	1.67	0.006	− 0.93	0.61	0.126
Mg	− 16.62	2.94	0.000	4.11	0.59	0.000	7.98	3.16	0.012	− 1.20	0.63	0.057
Mn	− 4.82	2.29	0.035	3.90	0.57	0.000	2.53	2.61	0.333	− 0.95	0.60	0.114
Ni	0.63	1.57	0.689	3.88	0.56	0.000	− 1.99	1.81	0.270	− 0.89	0.61	0.141
Zn	− 3.10	1.19	0.009	4.04	0.58	0.000	1.59	1.35	0.238	− 1.07	0.62	0.086
pH	− 8.74	7.12	0.220	− 1.44	1.16	0.217	2.73	13.04	0.834	0.44	2.20	0.842
*B*. *calyciflorus*												
	*β*_0_	*SE*_*β*0_	p_β0_	*β*_1_	*SE*_*β*1_	*p*_*β*1_	*β*_2_	*SE*_*β*2_	*p*_β2_	*β*_3_	*SE*_*β*3_	*p*_*β*3_
Al	− 0.60	0.65	0.357	1.28	0.40	0.002	0.48	0.78	0.536	0.317	0.500	0.525
As	0.21	0.97	0.829	0.84	0.33	0.011	− 0.08	1.13	0.943	0.991	0.439	0.024
Cr	− 0.45	1.25	0.715	1.22	0.38	0.001	− 0.54	1.45	0.709	0.342	0.463	0.460
Cu	0.65	0.76	0.391	1.22	0.39	0.002	− 1.64	0.87	0.059	0.523	0.485	0.281
Mg	− 5.80	1.94	0.003	1.37	0.42	0.001	1.11	2.16	0.609	0.193	0.514	0.708
Mn	− 1.98	1.23	0.109	1.27	0.44	0.004	0.70	1.40	0.616	0.311	0.501	0.535
Ni	− 0.06	0.92	0.950	1.28	0.40	0.001	− 0.69	1.07	0.518	0.406	0.494	0.411
Zn	− 1.28	0.87	0.138	1.33	0.41	0.001	0.44	0.99	0.659	0.343	0.508	0.500
pH	− 1.58	3.77	0.676	− 0.15	0.61	0.802	6.62	6.94	0.341	1.05	1.17	0.369

Excluding H, the influence of the elements’ concentration was similar to that of the test organism. The *β*_1_ parameter represents changes in the mortality of the test organisms with the concentration of the element. A higher sensitivity of *D*. *magna* (*β*_1_ range 3.40–4.11) than *B*. *calyciflorus* (*β*_1_ range 0.84–1.33) on the elements’ concentration in solution was observed, which reflected the obtained values of LC 50 for both test species (i.e., the lower toxicity to *B*. *calyciflorus*) during the ecotoxicity tests. The *p*_β3_ values for *D*. *magna* indicated no statistically significant differences between slopes *β*_1_ in the concentration–mortality relationships for the elements determined in the Wrocław and Katowice regions. For As and *B. calyciflorus*, a statistically significant difference β_3_ between slopes β_1_ for Wrocław and Katowice agglomerations was found, suggesting that this element can play a significant role in the contamination of URD. Therefore, As can negatively impact water organisms. Furthermore, for Cu and Mg, the differences in the intercepts in the relationships studied were statistically insignificant (*p*_*β*2_) for *D. magna*. For B*. calyciflorus*, no statistically significant differences (*p*_*β*2_) in the intercepts corresponding to the Wrocław and Katowice regions were found. For H (pH), no statistically significant differences with respect to the test organism and the sampling area were observed.

According to the authors, the similar characteristics of the elemental concentrations during the test of organism mortality for each chemical element suggest that there is no specific action related to the ion type. The synergistic effect of diverse elements may be influential in creating toxicity for the test organisms. The organisms’ mortality changed with respect to the element concentration rather than the element type. This observation may also indicate the coexistence of the elements analyzed in our studies with the unknown compound (or compounds) responsible for the test organisms’ mortality. This hypothesis is supported by PCA analysis of the total content of URD in the studied areas and the finding that the additional component PC2w, which is weakly or negatively correlated with any PCxs, appears in winter.

A statistically significant correlation between the total elements concentration in URD and the WS fraction indicates good solubility of the chemical compound containing the chemical element considered. Good solubility in water of a chemical compound enhances the bioavailability of its components, including chemical elements, which increases its negative influence on water organisms.

The results showed differences between the concentrations of elements in URD collected during summer and winter in both regions. For Zn, As, and Mg, significant correlations (*p*_*S*_ < 0.05) during winter were noticed. In samples collected during summer for Mn, As, and Mg, statistically significant correlations were observed. These results imply differences in the properties of the chemical compounds containing Zn and Mn during summer and winter. The Zn compounds are considered insoluble in water during summer, but Zn-containing compounds are WS in winter. In contrast to Zn, the Mn-containing WS compounds are expected to appear in summer, and the soluble compounds containing As and Mg appear in summer and winter. However, in general, it cannot be clearly stated that element concentration in simulated URD runoff is greater in the summer and winter months. Some elements are particularly more available in warm temperatures, while others are more available in low temperatures. As temperature affects air movement, it can also affect the composition of pollutants in URD leachate. Traffic pollutants increase in the cold season, and the risk of corrosion due to exposure to salt and water is also much higher in winter, contributing to the higher level of certain elements in URD runoff than in summer. However, this does not necessarily mean that there are more pollutants in winter. It is worth mentioning that industrial emissions remain the same all year round, while car exhaust fumes, the risk of corrosion, and fuel burning tend to increase during the winter months. In this study, the observed differences in winter and summer mortality of *D*. *magna* and *B*. *calyciflorus* confirm the negative impact of winter URD simulated runoff on organisms and/or the possible coexistence of the unknown compound (or compounds) responsible for their mortality.

To summarize, pollutants in URD are a common concern for urban areas due to their availability and tendency to bioaccumulate in living organisms. If the level of these elements exceeds their tolerance limit, it may result in toxicity of URD and directly influence the surface runoff during precipitation. This study presents a comprehensive evaluation of the seasonal toxicity of URD in the simulated runoff process. The results are helpful for researchers, policymakers and public health authorities when developing strategies to protect agglomerations against pollution from URD and protect residents’ health. Further studies should focus on the impact of toxic compounds/elements and should be extended, not only based on studies with simulated runoff but also on real dust runoff collected at study sites which also takes into account other compounds and toxic substances especially new emerging pollutants but should also be more comprehensive, i.e., based on a greater number of sites to allow identify relationships and significant patterns affecting the toxicity of waters and aquatic organisms on a large scale.

## Conclusions

This study found a statistically significant correlation between the concentration of total elements in URD and the WS fraction, indicating good solubility of the studied chemical elements. Concentration of Mn, Cu, Zn, and As in URD samples collected from the Katowice agglomeration was higher than that in URD from the Wrocław agglomeration which is mainly attributed to combustion processes (traffic and municipal origin), industrial and construction activities. The WS fraction values in URD were the highest for Mn, Zn, As, Cr, and, Mg for both summer and winter in the Upper Silesia region. Seasonal differences lead to higher levels of Zn, As, Cr, and Al in the WS fraction of URD during winter. Significant correlations were found between concentrations of Zn, As, and Mg also during winter. The highest mortality rates of *D*. *magna* and *B*. *calyciflorus* were also observed in winter URD.

This study indicates that the seasonal pollution in the analyzed areas exhibits seasonality due to the concentration of the studied elements and the possible influence of other factors. The data also revealed that large agglomerations, such as the Katowice and Wrocław regions, can greatly influence water quality through surface runoff. Therefore, it is necessary to take measures to prevent pollution leakage in such areas and protect water organisms. It is evident that industrialization and URD contamination resulting from combustion processes related to human activities have led to the ecosystem’s deterioration. This study highlights the alarming seasonal sources of elements in URD runoff, which will directly enter the food chain and affect the entire ecosystem. This work enhances our understanding of URD’s ecological risks.

### Supplementary Information

Below is the link to the electronic supplementary material.

Supplementary file1 (DOCX 70 KB)

## Data Availability

All data supporting the findings of this study are available within the paper and its Supplementary Information.
