# Supplementary material for: Seasonal toxicity of urban road dust in runoff process-studies in Poland
Source: Environ Sci Pollut Res Int. 2024 May 28;31(26):38485–99. doi: 10.1007/s11356-024-33716-w (PMC11189338; doi:10.1007/s11356-024-33716-w)
Supplement: Supplementary file 1 — Supplementary file1 (DOCX 70 KB) [file 11356_2024_33716_MOESM1_ESM.docx]

Supplementary material

**Seasonal toxicity of urban road dust in runoff process-studies in Poland**

**Justyna Rybak, Zbigniew Ziembik_,_ Magdalena Wróbel, Jan Bihałowicz, Wioletta Rogula-Kozłowska, Niranjala Dissanayake Mudiyanselage, Grzegorz Majewski**

Corresponding author: Justyna Rybak email: [justyna.rybak@pwr.edu.pl](mailto:justyna.rybak@pwr.edu.pl)

Telephone: +48713202528

Table S1. pH values for water extracts of URD.

| **sample ID** | **pH** | |
| --- | --- | --- |
|  | **summer** | **winter** |
| KAT1 | 7.8 | 6.8 |
| KAT2 | 6.4 | 6 |
| KAT3 | 6.2 | 6.1 |
| WRO1 | 6.3 | 6.1 |
| WRO2 | 7.1 | 7 |
| WRO3 | 6.8 | 7.2 |
| WRO4 | 6 | 6.4 |
| WRO5 | 6.2 | 6 |
| WRO6 | 6.4 | 6.2 |
| WRO7 | 7.2 | 7 |
| WRO8 | 7.5 | 7.3 |
| WRO9 | 6.8 | 6.4 |

Table S2. Description of sampling sites.

|  | Geographical coordinates | Location | Description | Traffic volume  $\frac{number of vehicles}{hour}$ |
| --- | --- | --- | --- | --- |
| KAT1 | 50°20'31.4"N 18°56'51.4"E | Bytom | The site is located outside the city, low-rise buildings and individual heating dominate, the area by the national road 94, several single-family houses and factories nearby | 660 |
| KAT2 | 50°20'44.4"N 18°58'02.5"E | Piekary Śląskie | The site is located in post-industrial area with abandoned hotels and wastelands, about 1.5 km from the old town with low-rise buildings characterised by individual heating, the area is located by the national road no. 94 | 520 |
| KAT3 | 50°20'17.3"N 18°58'28.6"E | Piekary Śląskie | The site is located about 1 km from the city center in the Brzeziny Śląskie district, surrounded by old and low-rise multifamily buildings with individual heating, the site is located by the national road no. 94 | 570 |
| WRO1 | 51°08'44.9"N 17°01'20.7"E | Wrocław, Obornicka street | The site is located next to a housing estate with compact and high-rise buildings with district heating. It is the exit route from the city towards Poznań | 300 |
| WRO2 | 51°09'56.2"N 17°09'37.1"E | Mirków | The village located north-east of Wroclaw, about 12 km from the city, along national road No. 98. Compact single-family housing with individual heating dominanates. | 100 |
| WRO3 | 51°06'28.1"N 17°02'24.9"E | Wrocław, Oławska street | The site in the very center of the city near the old market square. Compact housing development with district heating. The point is located at the most crowded place in the city - the old market square. | 400 |
| WRO4 | 51°07'14.4"N 16°59'41.6"E | Wrocław, Legnicka street | The site is located in the west of the city, leading to the expressway, and there is one of the largest shopping centers in the city. Compact, high-rise housing with district heating.. | 800 |
| WRO5 | 51°02'55.5"N 16°57'01.6"E | Bielany Wrocławskie | The suburb of Wrocław is located south east of Wroclaw. This is the area where there are many factories and shopping centers. The site is located about 5 km from the city center, there is no compact housing development. | 500 |
| WRO6 | 51°06'33.9"N 17°03'07.6"E | Wrocław, Grunwaldzki Bridge | The site is located in the city center by the national road 94 towards Warsaw. This is in the very center of the city, there is a compact housing development with district heating. | 800 |
| WRO7 | 51°05'39.6"N 17°01'57.1"E | Wrocław, Gliniana street | The site is located in the south of the city by the national road 98, next to a compact residential development with district heating, near the largest sports and recreation center in the city - Aquapark (approx. 500 m from the site). | 300 |
| WRO8 | 51°02'46.5"N 17°07'09.5"E | Radwanice | The village located to the south-east from Wroclaw; surroundings: low-rise single-family houses with individual heating. The site is approximately 2.5 km from the eastern bypass of Wrocław | 200 |
| WRO9 | 51°09'03.1"N 16°57'00.2"E | Maślice | The north-west part of Wroclaw; surroundings: low-rise single-family houses with individual heating, | 250 |

Table S3. Solubility in URD in summer samples [%]

| **Sample ID** | Mn-S | Ni-S | Cu- S | Zn-S | As-S | Cr-S | Mg-S | Al-S |
| --- | --- | --- | --- | --- | --- | --- | --- | --- |
| KAT1 | 56 | 28 | 8 | 1 | 16 | 5 | 99 | 0.04 |
| KAT2 | 2 | 19 | 2 | 0.2 | 7 | 15 | 97 | 0.003 |
| KAT3 | 85 | 23 | 1 | 0.5 | 16 | 26 | 98 | 0.06 |
| WRO1 | 14 | 9 | 5 | 1 | 39 | 5 | 69 | 0.01 |
| WRO2 | 20 | 8 | 48 | 11 | 82 | 21 | 39 | 0.14 |
| WRO3 | 90 | 19 | 35 | 2 | 88 | 3 | 81 | 0.19 |
| WRO4 | 0 | 15 | 28 | 6 | 71 | 5 | 66 | 0.09 |
| WRO5 | 9 | 11 | 31 | 5 | 96 | 3 | 49 | 0.07 |
| WRO6 | 22 | 22 | 6 | 2 | 89 | 22 | 97 | 0.14 |
| WRO7 | 0 | 22 | 29 | 5 | 88 | 6 | 59 | 0.08 |
| WRO8 | 2 | 15 | 41 | 1 | 91 | 2 | 31 | 0.03 |
| WRO9 | 73 | 24 | 25 | 5 | 93 | 10 | 92 | 0.05 |

Table S4. Solubility in URD in winter samples [%]

| **Sample ID** | Mn-S | Ni-S | Cu- S | Zn-S | As-S | Cr-S | Mg-S | Al-S |
| --- | --- | --- | --- | --- | --- | --- | --- | --- |
| KAT1 | 55 | 22 | 6 | 1 | 13 | 3 | 91 | 0.04 |
| KAT2 | 2 | 15 | 1 | 0.1 | 6 | 13 | 92 | 0.003 |
| KAT3 | 82 | 19 | 1 | 0.4 | 15 | 21 | 93 | 0.06 |
| WRO1 | 13 | 5 | 4 | 1 | 37 | 3 | 78 | 0.01 |
| WRO2 | 19 | 6 | 38 | 9 | 78 | 18 | 42 | 0.14 |
| WRO3 | 72 | 17 | 31 | 1 | 77 | 3 | 87 | 0.19 |
| WRO4 | 0.1 | 15 | 26 | 6 | 69 | 4 | 90 | 0.09 |
| WRO5 | 6 | 9 | 28 | 5 | 90 | 1 | 56 | 0.07 |
| WRO6 | 18 | 18 | 4 | 2 | 81 | 21 | 99 | 0.14 |
| WRO7 | 0.2 | 19 | 27 | 4 | 81 | 5 | 79 | 0.08 |
| WRO8 | 2 | 15 | 38 | 1 | 90 | 2 | 32 | 0.04 |
| WRO9 | 71 | 22 | 22 | 4 | 90 | 9 | 78 | 0.05 |

Table S5. Total element concentrations in URD in summer samples [mg·kg^–1^].

| **Sample ID** | **Mn-T** | **Ni-T** | **Cu- T** | **Zn-T** | **As-T** | **Cr-T** | **Mg-T** | **Al-T** |
| --- | --- | --- | --- | --- | --- | --- | --- | --- |
| KAT1 | 1040±102 | 29±3 | 187±18 | 3201±327 | 136±13 | 61±6 | 15600±1450 | 9378±954 |
| KAT2 | 1950±198 | 59±5 | 458±43 | 9535±924 | 405±43 | 64±6 | 43600±4500 | 122248±12200 |
| KAT3 | 2984±276 | 33±3 | 284±22 | 4076±421 | 191±19 | 43±4 | 29300±2800 | 8026±839 |
| WRO1 | 248±25 | 47±4 | 97±9 | 140±13 | 2±0.2 | 59±5 | 6410±633 | 5023±502 |
| WRO2 | 247±26 | 102±8 | 51±5 | 84±8 | 5±0.4 | 67±5 | 11600±1100 | 4051±405 |
| WRO3 | 171±17 | 34±2 | 43±4 | 103±11 | 2±0.2 | 53±4 | 4600±420 | 3912±300 |
| WRO4 | 215±20 | 34±2 | 95±10 | 185±17 | 2±0.2 | 52±4 | 4290±410 | 4030±400 |
| WRO5 | 251±21 | 51±4 | 69±7 | 122±12 | 2±0.2 | 67±5 | 6290±608 | 5601±550 |
| WRO6 | 225±22 | 43±4 | 483±45 | 224±23 | 4±0.4 | 58±4 | 5830±570 | 4109±440 |
| WRO7 | 424±43 | 54±4 | 200±21 | 230±23 | 5±0.4 | 87±8 | 5050±500 | 5141±555 |
| WRO8 | 270±27 | 58±4 | 38±4 | 164±15 | 4±0.4 | 206±20 | 11400±1100 | 9159±843 |
| WRO9 | 267±25 | 30±2 | 56±5 | 121±12 | 3±0.3 | 39±4 | 4830±450 | 5175±520 |

Table S6.Total element concentrations in URD in winter samples [mg·kg^–1^].

| **Sample ID** | Mn-T | Ni-T | Cu- T | Zn-T | As-T | Cr-T | Mg-T | Al-T |
| --- | --- | --- | --- | --- | --- | --- | --- | --- |
| KAT1 | 70±7 | 3±0.3 | 6±0.6 | 6340±660 | 0.5±0.05 | 8±0.8 | 32500±3200 | 14000±1400 |
| KAT2 | 1024±100 | 39±4 | 47±0.4 | 11240±1100 | 2±0.2 | 390±39 | 83600±8500 | 245230±24500 |
| KAT3 | 772±70 | 29±2 | 156±15 | 5780±555 | 815±80 | 66±6 | 45140±4500 | 16540±1600 |
| WRO1 | 300±30 | 107±10 | 38±4 | 180±18 | 3±0.3 | 163±16 | 8294±829 | 1035±102 |
| WRO2 | 216±20 | 42±4 | 147±15 | 150±15 | 2±0.2 | 68±7 | 13032±1300 | 6504±650 |
| WRO3 | 368±30 | 61±6 | 130±13 | 205±20 | 3±0.3 | 104±10 | 5102±520 | 7403±750 |
| WRO4 | 191±20 | 33±3 | 33±3 | 248±20 | 2±0.2 | 68±7 | 6029±608 | 8605±860 |
| WRO5 | 248±23 | 45±4 | 71±7 | 242±20 | 8±0.8 | 126±12 | 7899±780 | 10508±1050 |
| WRO6 | 230±23 | 17±2 | 41±4 | 451±45 | 3±0.3 | 34±3 | 6320±630 | 6394±635 |
| WRO7 | 174±17 | 18±2 | 37±4 | 429±42 | 2±0.2 | 31±3 | 7043±700 | 8291±820 |
| WRO8 | 177±17 | 70±7 | 19±9 | 346±34 | 2±0.2 | 74±7 | 12430±1200 | 10503±1050 |
| WRO9 | 310±31 | 227±22 | 15±2 | 238±23 | 3±0.3 | 159±16 | 4278±420 | 8203±820 |

Table S7. Element concentrations in water soluble URD fraction in summer samples [mg·kg^–1^].

|  | Mn-WS | Ni-WS | Cu-WS | Zn-WS | As-WS | Cr-WS | Mg-WS | Al-WS |
| --- | --- | --- | --- | --- | --- | --- | --- | --- |
| KAT1 | 578±57 | 8±0.8 | 14±1 | 45±4 | 21±2 | 3±0.3 | 15406±1500 | 4±0.4 |
| KAT2 | 44±4 | 11±1 | 9±0.9 | 17±2 | 28±2 | 9±0.9 | 42433±4200 | 4±0.4 |
| KAT3 | 2540±250 | 8±0.8 | 4±0.4 | 19±2 | 31±3 | 11±1 | 28727±2800 | 5±0.5 |
| WRO1 | 34±3 | 4±0.4 | 5±0.5 | 2±0.2 | 1±0.1 | 3±0.3 | 4395±430 | 1±0.1 |
| WRO2 | 50±5 | 9±0.9 | 24±2 | 9±0.9 | 4±0.4 | 14±1 | 4539±450 | 6±0.6 |
| WRO3 | 154±15 | 7±0.7 | 15±2 | 2±0.2 | 2±0.2 | 2±0.2 | 3711±370 | 7±0.7 |
| WRO4 | 1±0.1 | 5±0.5 | 26±2 | 11±1 | 2±0.2 | 3±0.3 | 2839±280 | 4±0.4 |
| WRO5 | 21±2 | 6±0.6 | 21±2 | 7±0.7 | 2±0.2 | 2±0.2 | 3074±300 | 4±0.4 |
| WRO6 | 49±5 | 9±0.9 | 28±2 | 5±0.5 | 3±0.3 | 13±1 | 5642±560 | 6±0.6 |
| WRO7 | 2±0.2 | 12±1 | 58±5 | 12±1 | 4±0.4 | 5±0.5 | 3005±300 | 4±0.4 |
| WRO8 | 6±0.6 | 9±0.9 | 15±2 | 2±­0.2 | 4±0.4 | 4±0.4 | 3510±350 | 3±0.3 |
| WRO9 | 196±19 | 7±0.7 | 14±1 | 6±0.6 | 3±0.3 | 4±0.4 | 4441±440 | 3±0.3 |

Table S8. Element concentrations in water soluble URD fraction in winter samples [mg·kg^–1^].

|  | Mn-WS | Ni-WS | Cu-WS | Zn-WS | As-WS | Cr-WS | Mg-WS | Al-WS |
| --- | --- | --- | --- | --- | --- | --- | --- | --- |
| KAT1 | 38±4 | 1±0.1 | 0.4±0.04 | 57±5 | 0.1±0.01 | 0.3±0.03 | 29413±28000 | 6±0.6 |
| KAT2 | 15±2 | 6±0.6 | 1±0.1 | 11±1 | 0.1±0.01 | 50±5 | 77246±7700 | 8±0.8 |
| KAT3 | 633±63 | 5±0.5 | 2±0.2 | 23±2 | 118±11 | 14±1 | 42161±4200 | 11±1 |
| WRO1 | 39±4 | 6±0.6 | 1±0.1 | 1±0.1 | 1±0.1 | 6±0.6 | 5018±500 | 0±0.0 |
| WRO2 | 40±4 | 3±0.3 | 56±5 | 13±1 | 1±0.1 | 13±1 | 4861±480 | 9±0.9 |
| WRO3 | 266±26 | 10±1 | 40±4 | 3±0.3 | 2±0.2 | 3±0.3 | 4005±400 | 14±1 |
| WRO4 | 0.2±0.02 | 5±0.5 | 8±0.8 | 14±1 | 1±0.1 | 3±0.3 | 3877±380 | 8±0.8 |
| WRO5 | 14±1 | 4±0.4 | 20±2 | 12±1 | 7±0.7 | 2±0.2 | 3547±350 | 7±0.7 |
| WRO6 | 42±4 | 3±0.3 | 2±0.2 | 10±1 | 2±0.2 | 7±0.7 | 5764±570 | 9±0.9 |
| WRO7 | 0.3±0.03 | 3±0.3 | 10±1 | 18±2 | 2±0.2 | 1±0.1 | 3993±390 | 7±0.7 |
| WRO8 | 4±0.4 | 10±1 | 7±0.7 | 3±0.3 | 2±0.2 | 1±0.1 | 3592±350 | 4±0.4 |
| WRO9 | 221±22 | 50±5 | 3±0.3 | 9±0.9 | 3±0.3 | 14±1 | 3760±370 | 4±0.4 |

Supplementary material

**Seasonal toxicity of urban road dust in runoff process-studies in Poland**

**Justyna Rybak, Zbigniew Ziembik_,_ Magdalena Wróbel, Jan Bihałowicz, Wioletta Rogula-Kozłowska, Niranjala Dissanayake Mudiyanselage, Grzegorz Majewski**

Corresponding author: Justyna Rybak email: [justyna.rybak@pwr.edu.pl](mailto:justyna.rybak@pwr.edu.pl)

Telephone: +48713202528

Table S1. pH values for water extracts of URD.

| **sample ID** | **pH** | |
| --- | --- | --- |
|  | **summer** | **winter** |
| KAT1 | 7.8 | 6.8 |
| KAT2 | 6.4 | 6 |
| KAT3 | 6.2 | 6.1 |
| WRO1 | 6.3 | 6.1 |
| WRO2 | 7.1 | 7 |
| WRO3 | 6.8 | 7.2 |
| WRO4 | 6 | 6.4 |
| WRO5 | 6.2 | 6 |
| WRO6 | 6.4 | 6.2 |
| WRO7 | 7.2 | 7 |
| WRO8 | 7.5 | 7.3 |
| WRO9 | 6.8 | 6.4 |

Table S2. Description of sampling sites.

|  | Geographical coordinates | Location | Description | Traffic volume  $\frac{number of vehicles}{\mathrm{hour}}$ |
| --- | --- | --- | --- | --- |
| KAT1 | 50°20'31.4"N 18°56'51.4"E | Bytom | The site is located outside the city, low-rise buildings and individual heating dominate, the area by the national road 94, several single-family houses and factories nearby | 660 |
| KAT2 | 50°20'44.4"N 18°58'02.5"E | Piekary Śląskie | The site is located in post-industrial area with abandoned hotels and wastelands, about 1.5 km from the old town with low-rise buildings characterised by individual heating, the area is located by the national road no. 94 | 520 |
| KAT3 | 50°20'17.3"N 18°58'28.6"E | Piekary Śląskie | The site is located about 1 km from the city center in the Brzeziny Śląskie district, surrounded by old and low-rise multifamily buildings with individual heating, the site is located by the national road no. 94 | 570 |
| WRO1 | 51°08'44.9"N 17°01'20.7"E | Wrocław, Obornicka street | The site is located next to a housing estate with compact and high-rise buildings with district heating. It is the exit route from the city towards Poznań | 300 |
| WRO2 | 51°09'56.2"N 17°09'37.1"E | Mirków | The village located north-east of Wroclaw, about 12 km from the city, along national road No. 98. Compact single-family housing with individual heating dominanates. | 100 |
| WRO3 | 51°06'28.1"N 17°02'24.9"E | Wrocław, Oławska street | The site in the very center of the city near the old market square. Compact housing development with district heating. The point is located at the most crowded place in the city - the old market square. | 400 |
| WRO4 | 51°07'14.4"N 16°59'41.6"E | Wrocław, Legnicka street | The site is located in the west of the city, leading to the expressway, and there is one of the largest shopping centers in the city. Compact, high-rise housing with district heating.. | 800 |
| WRO5 | 51°02'55.5"N 16°57'01.6"E | Bielany Wrocławskie | The suburb of Wrocław is located south east of Wroclaw. This is the area where there are many factories and shopping centers. The site is located about 5 km from the city center, there is no compact housing development. | 500 |
| WRO6 | 51°06'33.9"N 17°03'07.6"E | Wrocław, Grunwaldzki Bridge | The site is located in the city center by the national road 94 towards Warsaw. This is in the very center of the city, there is a compact housing development with district heating. | 800 |
| WRO7 | 51°05'39.6"N 17°01'57.1"E | Wrocław, Gliniana street | The site is located in the south of the city by the national road 98, next to a compact residential development with district heating, near the largest sports and recreation center in the city - Aquapark (approx. 500 m from the site). | 300 |
| WRO8 | 51°02'46.5"N 17°07'09.5"E | Radwanice | The village located to the south-east from Wroclaw; surroundings: low-rise single-family houses with individual heating. The site is approximately 2.5 km from the eastern bypass of Wrocław | 200 |
| WRO9 | 51°09'03.1"N 16°57'00.2"E | Maślice | The north-west part of Wroclaw; surroundings: low-rise single-family houses with individual heating, | 250 |

Table S3. Solubility in URD in summer samples [%]

| **Sample ID** | Mn-S | Ni-S | Cu- S | Zn-S | As-S | Cr-S | Mg-S | Al-S |
| --- | --- | --- | --- | --- | --- | --- | --- | --- |
| KAT1 | 56 | 28 | 8 | 1 | 16 | 5 | 99 | 0.04 |
| KAT2 | 2 | 19 | 2 | 0.2 | 7 | 15 | 97 | 0.003 |
| KAT3 | 85 | 23 | 1 | 0.5 | 16 | 26 | 98 | 0.06 |
| WRO1 | 14 | 9 | 5 | 1 | 39 | 5 | 69 | 0.01 |
| WRO2 | 20 | 8 | 48 | 11 | 82 | 21 | 39 | 0.14 |
| WRO3 | 90 | 19 | 35 | 2 | 88 | 3 | 81 | 0.19 |
| WRO4 | 0 | 15 | 28 | 6 | 71 | 5 | 66 | 0.09 |
| WRO5 | 9 | 11 | 31 | 5 | 96 | 3 | 49 | 0.07 |
| WRO6 | 22 | 22 | 6 | 2 | 89 | 22 | 97 | 0.14 |
| WRO7 | 0 | 22 | 29 | 5 | 88 | 6 | 59 | 0.08 |
| WRO8 | 2 | 15 | 41 | 1 | 91 | 2 | 31 | 0.03 |
| WRO9 | 73 | 24 | 25 | 5 | 93 | 10 | 92 | 0.05 |

Table S4. Solubility in URD in winter samples [%]

| **Sample ID** | Mn-S | Ni-S | Cu- S | Zn-S | As-S | Cr-S | Mg-S | Al-S |
| --- | --- | --- | --- | --- | --- | --- | --- | --- |
| KAT1 | 55 | 22 | 6 | 1 | 13 | 3 | 91 | 0.04 |
| KAT2 | 2 | 15 | 1 | 0.1 | 6 | 13 | 92 | 0.003 |
| KAT3 | 82 | 19 | 1 | 0.4 | 15 | 21 | 93 | 0.06 |
| WRO1 | 13 | 5 | 4 | 1 | 37 | 3 | 78 | 0.01 |
| WRO2 | 19 | 6 | 38 | 9 | 78 | 18 | 42 | 0.14 |
| WRO3 | 72 | 17 | 31 | 1 | 77 | 3 | 87 | 0.19 |
| WRO4 | 0.1 | 15 | 26 | 6 | 69 | 4 | 90 | 0.09 |
| WRO5 | 6 | 9 | 28 | 5 | 90 | 1 | 56 | 0.07 |
| WRO6 | 18 | 18 | 4 | 2 | 81 | 21 | 99 | 0.14 |
| WRO7 | 0.2 | 19 | 27 | 4 | 81 | 5 | 79 | 0.08 |
| WRO8 | 2 | 15 | 38 | 1 | 90 | 2 | 32 | 0.04 |
| WRO9 | 71 | 22 | 22 | 4 | 90 | 9 | 78 | 0.05 |

Table S5. Total element concentrations in URD in summer samples [mg·kg^–1^].

| **Sample ID** | **Mn-T** | **Ni-T** | **Cu- T** | **Zn-T** | **As-T** | **Cr-T** | **Mg-T** | **Al-T** |
| --- | --- | --- | --- | --- | --- | --- | --- | --- |
| KAT1 | 1040±102 | 29±3 | 187±18 | 3201±327 | 136±13 | 61±6 | 15600±1450 | 9378±954 |
| KAT2 | 1950±198 | 59±5 | 458±43 | 9535±924 | 405±43 | 64±6 | 43600±4500 | 122248±12200 |
| KAT3 | 2984±276 | 33±3 | 284±22 | 4076±421 | 191±19 | 43±4 | 29300±2800 | 8026±839 |
| WRO1 | 248±25 | 47±4 | 97±9 | 140±13 | 2±0.2 | 59±5 | 6410±633 | 5023±502 |
| WRO2 | 247±26 | 102±8 | 51±5 | 84±8 | 5±0.4 | 67±5 | 11600±1100 | 4051±405 |
| WRO3 | 171±17 | 34±2 | 43±4 | 103±11 | 2±0.2 | 53±4 | 4600±420 | 3912±300 |
| WRO4 | 215±20 | 34±2 | 95±10 | 185±17 | 2±0.2 | 52±4 | 4290±410 | 4030±400 |
| WRO5 | 251±21 | 51±4 | 69±7 | 122±12 | 2±0.2 | 67±5 | 6290±608 | 5601±550 |
| WRO6 | 225±22 | 43±4 | 483±45 | 224±23 | 4±0.4 | 58±4 | 5830±570 | 4109±440 |
| WRO7 | 424±43 | 54±4 | 200±21 | 230±23 | 5±0.4 | 87±8 | 5050±500 | 5141±555 |
| WRO8 | 270±27 | 58±4 | 38±4 | 164±15 | 4±0.4 | 206±20 | 11400±1100 | 9159±843 |
| WRO9 | 267±25 | 30±2 | 56±5 | 121±12 | 3±0.3 | 39±4 | 4830±450 | 5175±520 |

Table S6.Total element concentrations in URD in winter samples [mg·kg^–1^].

| **Sample ID** | Mn-T | Ni-T | Cu- T | Zn-T | As-T | Cr-T | Mg-T | Al-T |
| --- | --- | --- | --- | --- | --- | --- | --- | --- |
| KAT1 | 70±7 | 3±0.3 | 6±0.6 | 6340±660 | 0.5±0.05 | 8±0.8 | 32500±3200 | 14000±1400 |
| KAT2 | 1024±100 | 39±4 | 47±0.4 | 11240±1100 | 2±0.2 | 390±39 | 83600±8500 | 245230±24500 |
| KAT3 | 772±70 | 29±2 | 156±15 | 5780±555 | 815±80 | 66±6 | 45140±4500 | 16540±1600 |
| WRO1 | 300±30 | 107±10 | 38±4 | 180±18 | 3±0.3 | 163±16 | 8294±829 | 1035±102 |
| WRO2 | 216±20 | 42±4 | 147±15 | 150±15 | 2±0.2 | 68±7 | 13032±1300 | 6504±650 |
| WRO3 | 368±30 | 61±6 | 130±13 | 205±20 | 3±0.3 | 104±10 | 5102±520 | 7403±750 |
| WRO4 | 191±20 | 33±3 | 33±3 | 248±20 | 2±0.2 | 68±7 | 6029±608 | 8605±860 |
| WRO5 | 248±23 | 45±4 | 71±7 | 242±20 | 8±0.8 | 126±12 | 7899±780 | 10508±1050 |
| WRO6 | 230±23 | 17±2 | 41±4 | 451±45 | 3±0.3 | 34±3 | 6320±630 | 6394±635 |
| WRO7 | 174±17 | 18±2 | 37±4 | 429±42 | 2±0.2 | 31±3 | 7043±700 | 8291±820 |
| WRO8 | 177±17 | 70±7 | 19±9 | 346±34 | 2±0.2 | 74±7 | 12430±1200 | 10503±1050 |
| WRO9 | 310±31 | 227±22 | 15±2 | 238±23 | 3±0.3 | 159±16 | 4278±420 | 8203±820 |

Table S7. Element concentrations in water soluble URD fraction in summer samples [mg·kg^–1^].

|  | Mn-WS | Ni-WS | Cu-WS | Zn-WS | As-WS | Cr-WS | Mg-WS | Al-WS |
| --- | --- | --- | --- | --- | --- | --- | --- | --- |
| KAT1 | 578±57 | 8±0.8 | 14±1 | 45±4 | 21±2 | 3±0.3 | 15406±1500 | 4±0.4 |
| KAT2 | 44±4 | 11±1 | 9±0.9 | 17±2 | 28±2 | 9±0.9 | 42433±4200 | 4±0.4 |
| KAT3 | 2540±250 | 8±0.8 | 4±0.4 | 19±2 | 31±3 | 11±1 | 28727±2800 | 5±0.5 |
| WRO1 | 34±3 | 4±0.4 | 5±0.5 | 2±0.2 | 1±0.1 | 3±0.3 | 4395±430 | 1±0.1 |
| WRO2 | 50±5 | 9±0.9 | 24±2 | 9±0.9 | 4±0.4 | 14±1 | 4539±450 | 6±0.6 |
| WRO3 | 154±15 | 7±0.7 | 15±2 | 2±0.2 | 2±0.2 | 2±0.2 | 3711±370 | 7±0.7 |
| WRO4 | 1±0.1 | 5±0.5 | 26±2 | 11±1 | 2±0.2 | 3±0.3 | 2839±280 | 4±0.4 |
| WRO5 | 21±2 | 6±0.6 | 21±2 | 7±0.7 | 2±0.2 | 2±0.2 | 3074±300 | 4±0.4 |
| WRO6 | 49±5 | 9±0.9 | 28±2 | 5±0.5 | 3±0.3 | 13±1 | 5642±560 | 6±0.6 |
| WRO7 | 2±0.2 | 12±1 | 58±5 | 12±1 | 4±0.4 | 5±0.5 | 3005±300 | 4±0.4 |
| WRO8 | 6±0.6 | 9±0.9 | 15±2 | 2±­0.2 | 4±0.4 | 4±0.4 | 3510±350 | 3±0.3 |
| WRO9 | 196±19 | 7±0.7 | 14±1 | 6±0.6 | 3±0.3 | 4±0.4 | 4441±440 | 3±0.3 |

Table S8. Element concentrations in water soluble URD fraction in winter samples [mg·kg^–1^].

|  | Mn-WS | Ni-WS | Cu-WS | Zn-WS | As-WS | Cr-WS | Mg-WS | Al-WS |
| --- | --- | --- | --- | --- | --- | --- | --- | --- |
| KAT1 | 38±4 | 1±0.1 | 0.4±0.04 | 57±5 | 0.1±0.01 | 0.3±0.03 | 29413±28000 | 6±0.6 |
| KAT2 | 15±2 | 6±0.6 | 1±0.1 | 11±1 | 0.1±0.01 | 50±5 | 77246±7700 | 8±0.8 |
| KAT3 | 633±63 | 5±0.5 | 2±0.2 | 23±2 | 118±11 | 14±1 | 42161±4200 | 11±1 |
| WRO1 | 39±4 | 6±0.6 | 1±0.1 | 1±0.1 | 1±0.1 | 6±0.6 | 5018±500 | 0±0.0 |
| WRO2 | 40±4 | 3±0.3 | 56±5 | 13±1 | 1±0.1 | 13±1 | 4861±480 | 9±0.9 |
| WRO3 | 266±26 | 10±1 | 40±4 | 3±0.3 | 2±0.2 | 3±0.3 | 4005±400 | 14±1 |
| WRO4 | 0.2±0.02 | 5±0.5 | 8±0.8 | 14±1 | 1±0.1 | 3±0.3 | 3877±380 | 8±0.8 |
| WRO5 | 14±1 | 4±0.4 | 20±2 | 12±1 | 7±0.7 | 2±0.2 | 3547±350 | 7±0.7 |
| WRO6 | 42±4 | 3±0.3 | 2±0.2 | 10±1 | 2±0.2 | 7±0.7 | 5764±570 | 9±0.9 |
| WRO7 | 0.3±0.03 | 3±0.3 | 10±1 | 18±2 | 2±0.2 | 1±0.1 | 3993±390 | 7±0.7 |
| WRO8 | 4±0.4 | 10±1 | 7±0.7 | 3±0.3 | 2±0.2 | 1±0.1 | 3592±350 | 4±0.4 |
| WRO9 | 221±22 | 50±5 | 3±0.3 | 9±0.9 | 3±0.3 | 14±1 | 3760±370 | 4±0.4 |
